# Supplementary material for: Primary tumor-induced immunity eradicates disseminated tumor cells in syngeneic mouse model
Source: Nat Commun. 2019 Mar 29;10:1430. doi: 10.1038/s41467-019-09015-1 (PMC6441000; doi:10.1038/s41467-019-09015-1)
Supplement: Supplementary file 1 — Supplementary Information [file 41467_2019_9015_MOESM1_ESM.pdf]

# Primary tumor-induced immunity eradicates disseminated tumor cells in syngeneic mouse model

Piranlioglu et al.

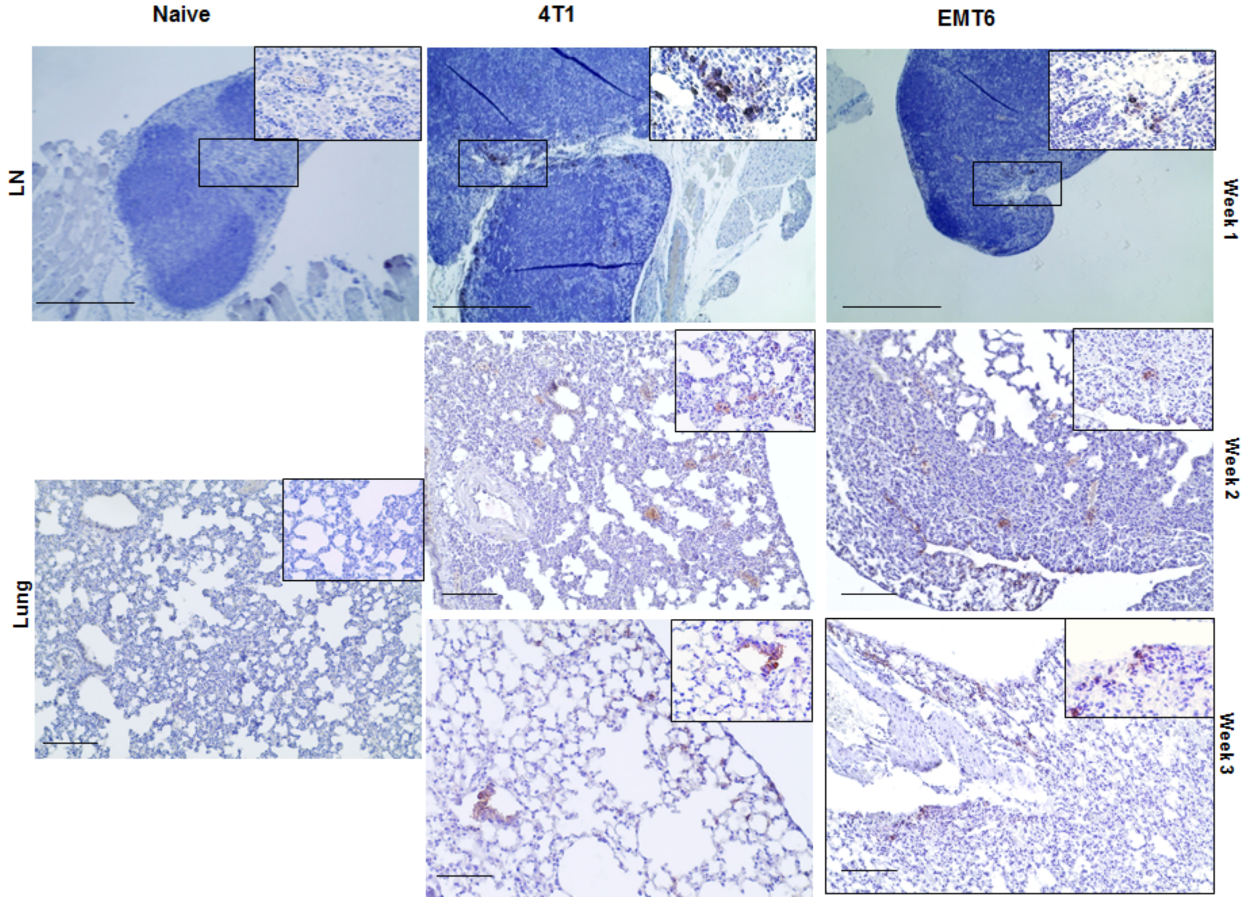


Supplementary Figure 1: Both metastatic 4T1 and non-metastatic EMT6 tumor disseminate secondary organs as early as week 1. Solitary disseminated tumor cells were detected at the LN and lungs of tumor-bearing mice at week1 and week 2 post - implantation, respectively. These DTCs grew into micro metastases in the lungs of 4T1 tumor-bearing mice at week3 while EMT6 tumor-bearing tumor had only solitary DTCs. Scale bar 100μm (LN) and 50μm(lungs).

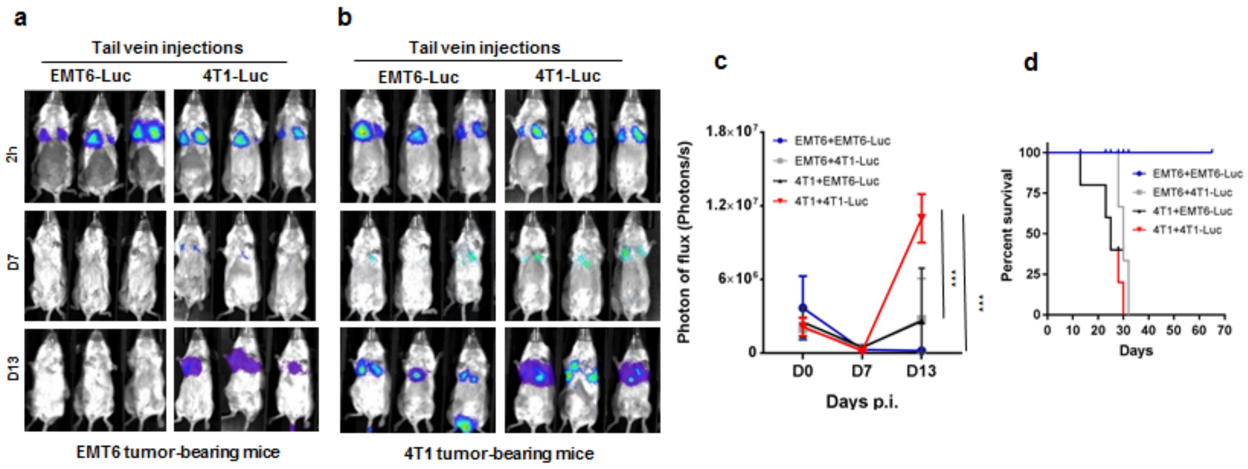


Supplementary Figure 2: 4T1 tumor primes a metastatic niche that promotes the metastatic outgrowth of IV-injected tumor cells in the lungs. (a-c) 50K parental tumor cells were implanted into 4$^{th}$ mammary fat pad of BALB/c mice. Primary tumors were resected 3 weeks post-implantation and luciferase-expressing tumor cells were injected through the tail vein one week after removal of tumors. 4T1 tumor-primed animals showed accelerated metastatic growth and shortened survival compared to EMT6 tumor-implanted mice when 4T1-Luc or EMT6-Luc tumor cells intravenously injected. Results are presented as mean± SD (5–10 mice in each group) ***$P$<0.0005 unpaired $t$-test.

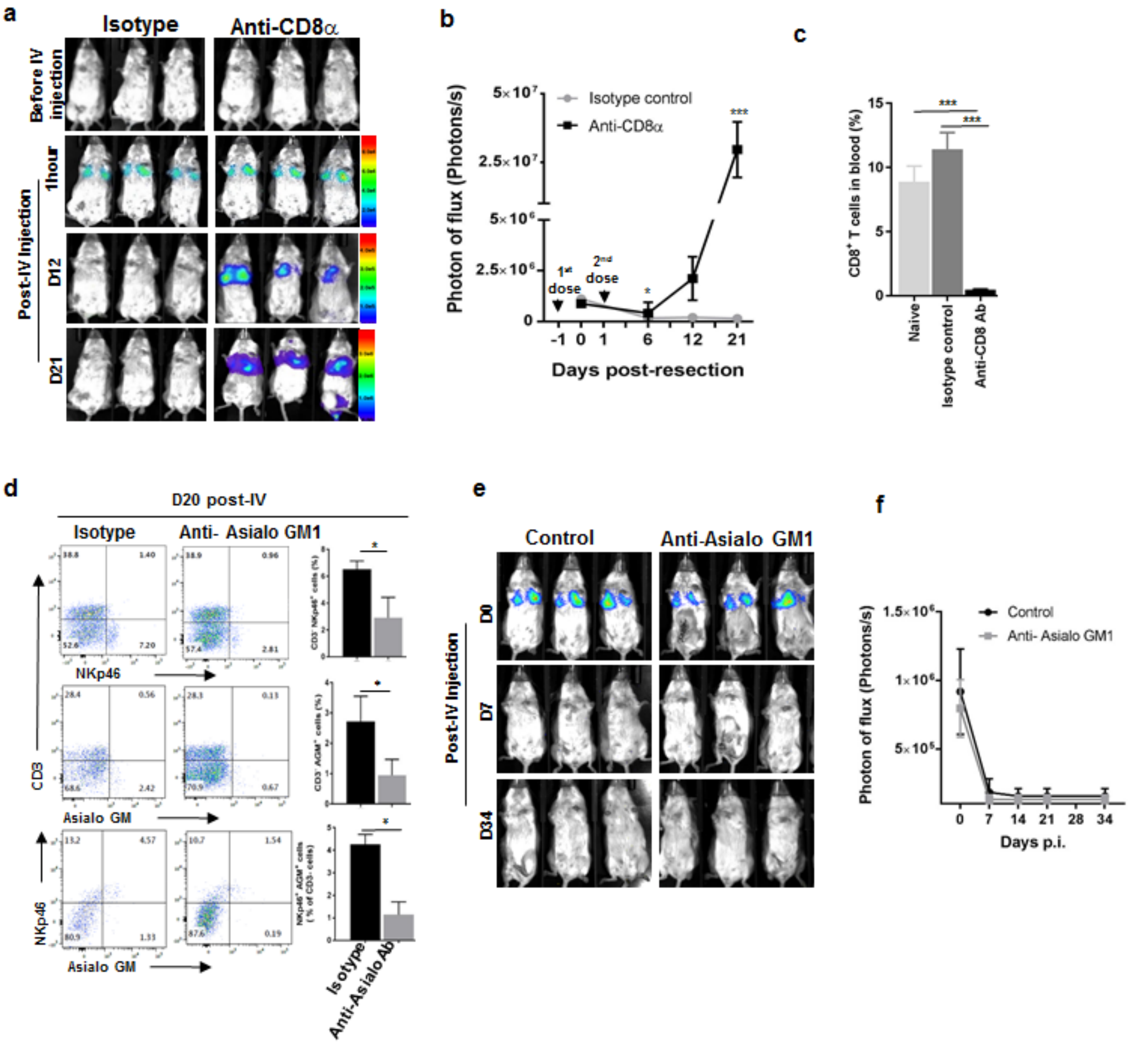


Supplementary Figure 3: CD8 T cell depletion results in metastatic growth while NK cell depletion has no effect. (a-c) EMT6 tumor-primed mice-treated with two doses of anti-CD8α antibody 6 months after resection of primary tumors supported the outgrowth of tail vein injected EMT6-Luc cells, while isotype treated mice effectively eliminated these cells. ***$P<0.0005$, one-way analysis of variance test. d-f, Treatment of EMT6-primed mice with 20μl of anti-Asialo GM antibody which effectively eliminates the NK cell population, had

no effect in elimination of tail vein injected EMT6-luc cells. NK cells were gated on $CD3^-$ lymphocyte population to check NKp46 and Anti-Asialo GM1 positive cells. 5-8 mice were used for each group. Flow results are presented as mean ±SD (n=3). *P<0.05, two-tailed Student's *t*-test.

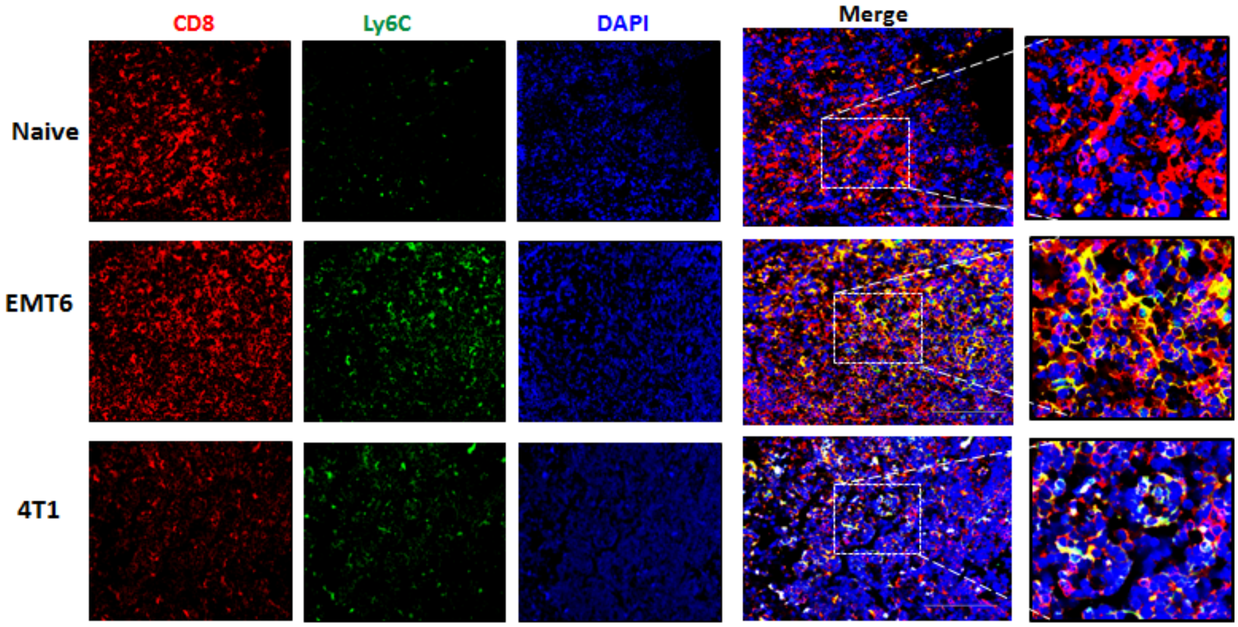


Supplementary Figure 4: In situ analyses of $Ly6C^+CD8^+$ T cells in axillary lymph nodes from naïve, EMT6 and 4T1 tumor-bearing mice. Lymph nodes from EMT6 tumor-primed mice show higher levels of $Ly6C^+CD8^+$ cells compared to those from naïve and 4T1 tumor-bearing mice at week 6 post-implantation of tumor cells into $4^{th}$ mammary fat pad of immunocompetent BALB/c mice.

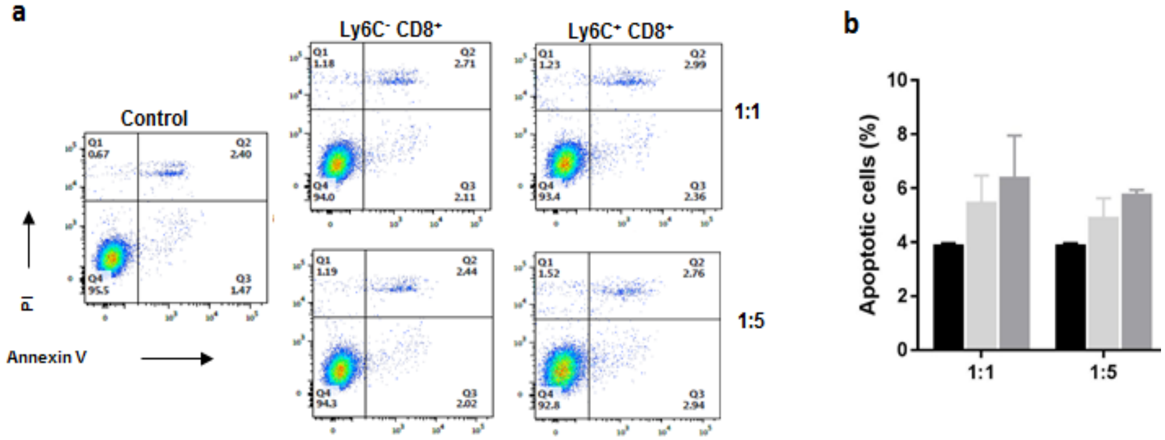


Supplementary Figure 3: Ly6C⁺ CD8⁺ T cells populations isolated from naïve (non-tumor bearing) mice failed to show cytotoxic activity against EMT6 tumor cells. (a-b) When co-cultured with EMT6 tumor cells in a ratio of 1:5 , $Ly6C^{+}CD8^{+}$ T cell population that were sorted from spleen of naïve mice did not induce a significant cell death compared to the $Ly6C^{-}CD8^{+}$ subset. Two-way analysis of variance test was used to compare groups. Results are presented as mean ±SD (n=3)
