# Supplementary material for: Primary tumor-induced immunity eradicates disseminated tumor cells in syngeneic mouse model
Source: Nat Commun. 2019 Mar 29;10:1430. doi: 10.1038/s41467-019-09015-1 (PMC6441000; doi:10.1038/s41467-019-09015-1)
Supplement: Supplementary file 4 — Description of Additional Supplementary Files [file 41467_2019_9015_MOESM4_ESM.docx]

**Description of Additional Supplementary Files**

File Name: Supplementary Data 1

Description: Gene expression analyses of LY6C+ EMT6-Lung vs LY6C+ 4T1-Lung. Mice bearing EMT6 or 4T1 tumors were necropsied at 3-week post-implantation and lung tissues were minced and digested with collagenase. Total RNA was isolated from single cells and were stained with isotype or anti-Ly6C antibody. The Ly6C+ cells were sorted by fluorescence-activated cell sorting (FACS) and subjected to mouse transcriptome analyses as described in the methods. Data is presented as mean value $\mp$SD (n=2).
